# “*The project did not come to us with a solution”:* Perspectives of research teams on implementing a study about electronic health record-embedded individualized pain plans for emergency department treatment of vaso-occlusive episodes in adults with sickle cell disease

**DOI:** 10.1186/s12913-023-10255-7

**Published:** 2023-11-13

**Authors:** Ana A. Baumann, Jane S. Hankins, Lewis L. Hsu, Robert W. Gibson, Lynne D. Richardson, Marsha Treadwell, Jeffrey A. Glassberg, Sarah Bourne, Lingzi Luo, Rita V. Masese, Terri Demartino, Judith Nocek, Elizabeth Taaffe, Sierra Gollan, Ome-Ollin Ruiz, Chinonyelum Nwosu, Nai Qashou, Aimee S. James, Paula Tanabe, Allison A. King

**Affiliations:** 1 Division of Public Health Sciences, Department of Surgery, Washington University, Saint Louis, MO USA; 2 Department of Global Pediatric Medicine, St. Jude Children’s Research Hospital, Memphis, TN USA; 3 Center for Sickle Cell Disease, University of Tennessee Health Science Center, Memphis, TN USA; 4 Sickle Cell Center, University of Illinois at Chicago, Chicago, IL USA; 5 Augusta University, Augusta, GA USA; 6 Icahn School of Medicine at Mount Sinai, Institute for Health Equity Research, New York City, NY USA; 7 Department of Pediatrics, Division of Hematology, University of California San Francisco, San Francisco, CA USA; 8 Department of Emergency Medicine, Icahn School of Medicine at Mount Sinai, New York, NY USA; 9 Addiction Sciences Division, Medical University of South Carolina, Charleston, SC USA; 10 School of Global Public Health, New York University, New York City, NY USA; 11 Duke University School of Nursing, Durham, NC USA; 12 Department of Pediatrics, Washington University in St. Louis, St Louis, MO USA; 13 Department of Hematology, St. Jude, Memphis, TN USA

**Keywords:** Sickle cell disease, Emergency department, Implementation science

## Abstract

**Background:**

This study aimed to capture the implementation process of the ALIGN Study, (An individualized Pain Plan with Patient and Provider Access for Emergency Department care of Sickle Cell Disease). ALIGN aimed to embed Individualized Pain Plans in the electronic health record (E-IPP) and provide access to the plan for both adult patients with sickle cell disease (SCD) and emergency department providers when a person with SCD comes to the emergency department in vaso-occlusive crises.

**Methods:**

Semi-structured interviews were conducted with research teams from the 8 participating sites from the ALIGN study. Seventeen participants (principal investigators and study coordinators) shared their perspectives about the implementation of ALIGN in their sites. Data were analyzed in three phases using open coding steps adapted from grounded theory and qualitative content analysis.

**Results:**

A total of seven overarching themes were identified: (1) the E-IPP structure (location and upkeep) and collaboration with the informatics team, (2) the role of ED champion, (3) the role of research coordinators, (4) research team communication, and communication between research team and clinical team, (5) challenges with the study protocol, (6) provider feedback: addressing over-utilizers, patient mistrust, and the positive feedback about the intervention, and (7) COVID-19 and its effects on study implementation.

**Conclusions:**

Findings from this study contribute to learning how to implement E-IPPs for adult patients with SCD in ED. The study findings highlight the importance of early engagement with different team members, a champion from the emergency department, study coordinators with different skills and enhancement of communication and trust among team members. Further recommendations are outlined for hospitals aiming to implement E-IPP for patients with SCD in ED.

**Supplementary Information:**

The online version contains supplementary material available at 10.1186/s12913-023-10255-7.

## Background

Sickle cell disease (SCD) is an inherited blood disorder affecting approximately 100,000 people in the United States [1], and millions worldwide. In the US, SCD predominantly affects African Americans and other underrepresented minorities. SCD results in a lifetime of anemia, severe pain, and end-organ damage [2], with the average survival of African American individuals with SCD approximately 30–45 years less than the average life expectancy for African Americans [1, 3].

The context of SCD care is complex and permeated with disparities. In addition to being members of a racial minority, the majority of people with SCD are socioeconomically disadvantaged and are less likely than their counterparts to be linked to quality systems of care [4–7]. Persons with SCD are at risk for life-threatening infections and strokes, among other complications [5], and have to be able to manage their disease, including emergency departments, observation units, and inpatient hospitalization [6], as well as primary care and specialty care, though the latter may not always be available. The severe pain – vaso-occlusive episodes – often require high doses of opioids to treat [8]. Managing SCD is particularly complicated as adolescents are transitioning into adulthood, when often they move to an adult provider and must take more responsibility for managing their disease complications [7].

Patients with SCD often require immediate treatment for their pain in emergency departments (EDs). EDs provide an important access to care for historically underserved populations; however inequities in care for people with SCD in the ED is complicated. Patients with SCD, particularly adult patients, often have considerably longer wait times in the ED compared to other patients to receive care [9], providers may not know how to prescribe opioids for this population [10] and patients report perceived discrimination from providers [11–13]. To address some of these issues, the National Heart, Lung and Blood Institute released a set of evidence-based recommendations for treating SCD (13) in 2014. The use of “an individualized prescribing and monitoring protocol or an SCD-specific protocol whenever possible” [14] in all settings was among the treatment recommendations for vaso-occlusive episodes. Nevertheless, the therapies proven to be efficacious are not reaching those in need [15, 16]. To address this gap, studies have found that the individualized pain plans (IPPs) decrease hospital admissions and readmissions, and improved pain scores for pediatric [17–19] and adult patients [20], although data for adult patients is still emerging [21, 22].

With the goal of improving the health and well-being of individuals ages 15–45 with SCD [23], the Sickle Cell Disease Implementation Consortium (SCDIC), a collaborative, multi-center research program, was established in 2016. Originally, each of the eight sites proposed different interventions, and we had proposed to capture the components of each intervention [24]. To assist in cross-site collaboration, the consortium developed interventions to be implemented across all sites focused in three main areas: (a) improving hydroxyurea use and adherence, (b) increasing pain management in the emergency department, and (c) reducing the number of unaffiliated patients (patients without a sickle cell health care provider) [25]. We then adapted the original study aims to characterize the strategies that each site used to implement their study protocol. Specifically, we report here data from interviews with study teams about the implementation process of the emergency department intervention: providing easy electronic access to individualized pain plans for emergency care in the electronic health record and to patients via the corresponding patient portal [25]. Before describing the aims of this current study, we will briefly explain the goals of the emergency department study.

### Overview of the emergency department study

**Fig. 1:**
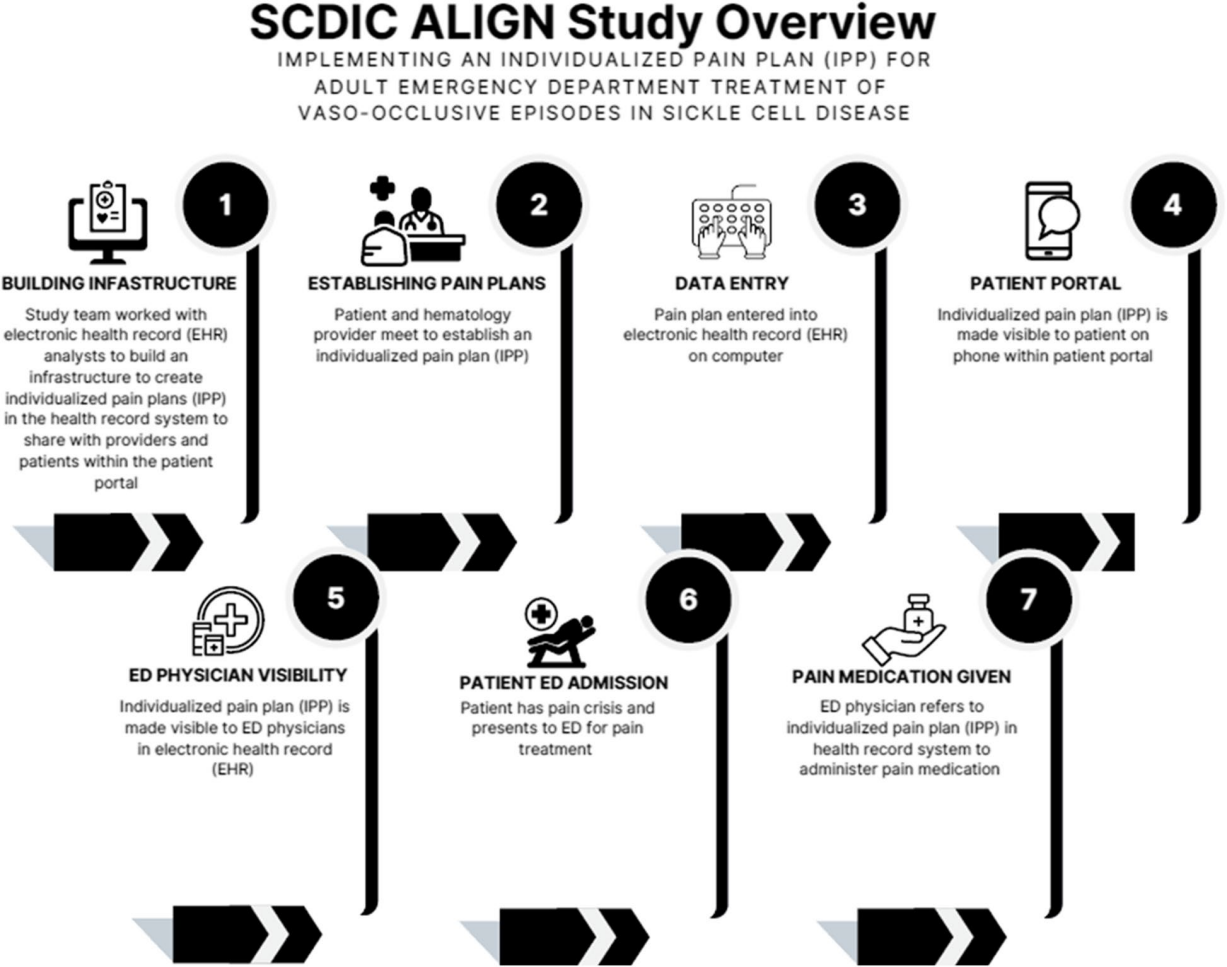
SCDIC Align Study Overview

The ALIGN Study, (An individualized Pain Plan with Patient and Provider Access for Emergency Department care of Sickle Cell Disease), has the overarching goal of implementing NHLBI recommendations for vasoocluse pain events (VOE) treatment by embedding Individualized Pain Plans in the electronic health record (E-IPP) and providing immediate access to the plan for both adult patients with SCD and emergency department providers when a person with SCD comes to the emergency department in vaso-occlusive crises. The intervention of the study consists of an E-IPP that contains the patient’s SCD genotype and analgesic medication recommendations developed by the SCD provider (See Fig. 1). The ALIGN study primarily aimed to examine the effectiveness of access to E-IPPs in improving patient and provider outcomes with pain treatment in the adult emergency department.

The E-IPPs were developed by the sickle cell providers at each study site, and each site collaborated with their informatics department to make the E-IPPs available to emergency providers via the provider electronic health record and to patients via the corresponding patient portal. To the best of our knowledge, this is the first time E-IPP’s have been available to the patient in the electronic health record for their access. To support the launch of the intervention, each site was required to do the following for enrolled patients: do a study orientation (install the electronic health record patient portal app, show a presentation demonstrating how to access the E-IPP, and do a teach-back method with the patients). In addition, patients at some institutions were provided with wallet cards that included instructions for the patient on how to access their E-IPP to allow them to show their pain plan to any ED provider in any health system. ED provider training involved a short video demonstrating where to find IPPs for study participants. The provider video also provided training on the prescribing of opioids and pain perception in sickle cell disease.

To be included in the ALIGN study, patients needed to have a confirmed diagnosis of SCD, speak English, be between the ages of 18–45 years old, have access to a mobile phone, have at least one vaso-occlusive episode visit to the site’s ED within the past 90 days prior to enrollment, and be willing to give informed consent. Further information about the intervention, inclusion and exclusion criteria, measures, designs and settings of this study can be found in Luo et al. (2021).[8]

### Understanding the infrastructure behind the study protocol

There is a high need to increase transparency and reproducibility of studies [26]. The lack of detailed descriptions of the interventions and the complexity entailed in implementing them is still prominent in the literature and may yield inefficient use of research efforts and dollars [27]. Describing complex interventions in healthcare systems is challenging, however, because of the dynamic interplay between interventions and the strategies that support their implementation [28]. Additionally, interventions and/or strategies may change from the moment that study protocols were published to the actual implementation of these interventions [24, 29]. The goal of the current study is to address such gaps by characterizing the implementation process of the ED intervention based on the perspective of different research team members from each site. The data from this study will support the development of the scale up intervention to other sites, and support dissemination and reproducibility by offering guidance to sites that aim to implement E-IPP to improve treatment delivery for people with SCD in emergency departments.

## Methods

### Study purpose and guiding research questions

Guided by the recent report by the National Academy of Engineering (NAE) and the Institute of Medicine (IOM) calling for applying systems engineering methods to healthcare systems [30], we focused on different levels in the team that are interdependent and necessary to improve the quality of care. This study is approved by the Washington University in St. Louis Human Research Protection Office (IRB Protocol # 201,709,005) and registered in the ClinicalTrial.Gov (#NCT03380351).

### Design

This is a qualitative study aimed at capturing details of the implementation process of a study.

### Setting

The study took place in the 8 SCDIC sites implementing the ALIGN Study. The emergency department practice patterns varied per site. Seven were academic sites, and one was a private hospital, and seven of the sites (including the private hospital) were urban, and one was suburban. More details about the sites can be found in Luo et al. [8]. All sites except two already had E-IPP’s in the electronic health record, (although not easily found by ED providers); none of the study sites had E-IPP’s visible to the patient.

### Participants

Participants for this study included the Principal Investigator (PI) of each of the participating sites and two study or nurse coordinators from each site, as indicated by the PIs. All participants consented for the interview.

### Data collection processes

All interviews were conducted via zoom from February to March of 2022 by AB individually with each participant. Participants were asked about how they were recruiting patients and providers to the study, what were some of the challenges and lessons learned from the study, and about communication among the team members (see Appendix 1 for the interview guide). Most interviews were conducted when the sites were either in the middle or towards the end of the implementation process, when the sites were reaching the targeted recruitment rates. Interviews lasted for about 45 min on average. Recorded interviews were professionally transcribed, deidentified and stored in a secure system.

### Data analysis

Data were analyzed in three phases using open coding steps adapted from grounded theory and qualitative content analysis [31]. First, Ana Baumann coded the interviews using open coding. Then, she developed a coding and recoded the interviews using such frame. Next, each participant received a sheet with the representative codes and quotes from their interview for member-checking. Once participants either edited their document and/or approved the document with their coded interview, AB further checked for de-identification and shared the coded data with Nai Qashou. NQ then reviewed the coding and, in an iterative fashion, compiled the themes of the interviews. Quotes for the paper were identified as being representative of the larger themes from the interviews. Several iterations of the paper were shared with participants for feedback on themes and representation of quotes.

### Reflexivity

The PI of this study is a Latina (born in Brazil), white skin, colonizer, able bodied, single mother in academia. Her area of research is in the intersection of implementation science, adaptation science and health equity. She has collaborated with several investigators in implementing evidence-based interventions in low-resource settings in the U.S. and globally, and in different areas of research (e.g., sickle cell, mental health, cancer). The study is part of a Diversity Supplement (PI: AB) funded by the National Heart, Lung and Blood Institute. The interview guide was co-developed with senior researchers members of the SCDIC Implementation Working Group, which included senior investigators experts in implementation science, sickle cell and qualitative research. The findings of the study were shared individually with each participant, and a draft of the paper was shared to the collective group of participants to enhance the trustworthiness and collaborative approach of the data.

## Results

Interviews were conducted with 17 participants (one site had only one participant, two sites had three participants and five sites had only two participants). A total of seven overarching themes were identified, described below.

### Theme 1: The E-IPP structure (location and upkeep) and collaboration with the informatics team.

A common struggle expressed by multiple sites was finding an appropriate location in the EHR/portal to place the IPPs as their visibility is important for effective care:

“*the challenge was to find a place that could be made visible in the [patient portal] because at that time, not all parts of the Epic record could actually be viewed in [patient portal]. (…) I don't think the technical details matter, but a place that's easy for the ED staff to get to and the patients can access it from [patient portal]. The place in the EHR where it is, it's actually a few clicks down. So we actually created what's called a best practice alert.”* (1W033_ED)

Building the IPP required close collaboration with the information technology department. Each site worked with their IT department to incorporate pain plans to their site’s electronic health record software. A few participants talked about their difficulties doing so:

*“there's a lot of intricacies with modifying another form and then making sure the right people can see it. So then it was getting the language that we wanted on it. And then where does it show up in the chart? And then how, if the patient has a pain plan, we expected to have a larger activation notice, a larger alert notice, but the IT people had recently created alert zone committee. (..) The hardest part, because we've had a couple of reorgs with IT, (…). And so nobody knows who does what.” (1W029_ED)*

*“Then, the next thing, for our site in particular, we did not have pain plans before, so it literally took me over two years to have the [informatics] team build this out (…). At one point, the head of the [electronic health record platform] thing told me that my analyst left, and I somehow got lost, but still, even, I think, if things have gone well, it probably would have taken me well over a year. It took over two years to get the build. A lot of our patients don’t have pain plans.” (1W023_ED P33)*

When asked what a new site would have to do to implement this intervention, a participant referred to the importance of early collaboration with the IT department:

*“Make sure you have the right IT person on board and able to move things along. Because had we had IT sorted, everything else would've rolled fairly quickly. So I think that's from an implementation perspective, besides the relationships between the providers and that type of thing. If your IT is not ready to set this up, or unwilling, or can't, that has been the greatest hindrance. Because again, the providers, no problem to recruit or train; patients, no problem to recruit or train. Getting everything else started was the hard part. So I think probably, if you have IT on board and are willing and able to do what you need, as long as everybody else gets along then you're in a good stead” (1W029_ED)*

In sites where pain plans have previously been in place, the problem of building and placing the pain plans was less prevalent. Their process seemed to be more efficient as those sites were able to utilize the template made for writing the pain plans and progress quicker with the implementation.

Sustaining the pain plans in the long term requires consistent upkeep and tracking. Hematologists were asked to update the IPP every six months. At some sites, this required rewriting a plan in a new document, while at others it was simply clicking a button in the EHR form to acknowledge the date reviewed. Participants identified the need for an efficient tracking system to ensure the plans are up-to-date and relevant to patients, which is a challenge considering other competing demands in the hematology clinic. In some cases, the direct hematology provider updated the plan and in others, the provider delegated the physical process to clinical staff who assisted the provider.

“*(..) I think the issue that we found with the pain plan concept is that it’s a little bit difficult to keep track of who’s got them, when they were put in, whether they need to be updated (…) the main issue will be, again, the updating of them. It’s all dependent on staffing. There’s many priorities to running a sickle cell program, and time that we spend updating pain plans could be time that we spend on making sure that everyone gets an echocardiogram.”* ﻿(1W032_ED)

Although each site had standardized its pain plans for its emergency department, the pain plans were not set as a standard of care in other hospitals. Some participants reported that patients' use of other hospitals interfered tracking the outcomes of all ED visits study since the pain plans are not visible in the outside EHR or followed by every emergency department in their area: “*not everyone comes to the three main hospitals for care, so how do you get pain plans written for them?”* (1W031_ED).

### Theme 2: The role of ED champion

The majority of sites recognized that buy-in from emergency department physicians and hematologists was crucial in making the implementation successful as they set the standard for treatment. When asked what helped implement the intervention at the provider level, participants answered:

*“We have a coinvestigator who's in emergency medicine physician who's the champion really for me. Without that, I don't think any site's going to be successful”. “There's a power differentiation between staff and faculty and knowledge too and just the ability to motivate the other ED physicians to do the training to complete the survey to get the education.” (1W025)*

### Theme 3: The role of research coordinators

While reflecting on the process of implementation, participants highlighted the role research coordinators played in the study. Coordinators have been responsible for i) screening the clinic schedule as a method of identifying patients eligible for the study; ii) enrolling and training participants; iii) working with sickle cell providers to customize the pain plans for study participants; and iv) data entry during the study. For this to be successful, coordinators had to work and communicate with clinical staff on a regular basis. This approach to recruitment was described in many of the participants’ interviews:

“*(…) and once a month, [the coordinator] runs a list of all the people who've been through the emergency department who have sickle cell disease. Then, we start with that list because part of the inclusion criteria is that you've been in within the past 90 days, and we start with the people who are the furthest out so closest to expiring for eligibility. (…). What's supposed to happen is if you go to the emergency department, you should have some kind of outpatient follow-up within a few weeks. We were hoping we could catch people that way. Then, if the patients didn't have a pain plan already built, then one of the coordinators reaches out. The coordinators then (…) look at the clinic list. See who's going up. If they patient is scheduled to be seen that week in the clinic, then the coordinators are prompting the provider. If there's not already a pain plan, we could they make sure that they build a pain plain and then confirm the eligibility and then try to enroll. You also have to make sure the patient has MyChart, but we've kinda figured that out.”* (P33)

*“I would send out certain providers list to the nurse coordinator who would be in the clinic with that provider's patient. For other providers, I will send out the email list to another study coordinator. [Coordinator] would send a blast email to the team. By the team, I mean the nurse coordinator, but we also have another coordinator to recruit for other studies because we want to be mindful of not sending the sickle cell providers too many emails for too many different studies. Then I send out these lists to the other coordinators so that she can compile the information for all of the studies that's going on.”*  (1W022_ED)

To ensure patients had access to their pain plans and are able to communicate with their provider, they must have access to the patient portal. Coordinators would ensure that patients had their portal and knew how to use it. Coordinators would try different methods and strategies to reach patients. Their persistence enabled successful data collection and tracking study progress:

“*I would say to make sure that you have a strong team in place with experience recruiting and someone for the data side of it too. Just to be persistent, too, and make sure that you're really exhausting all possible avenues of reaching out to someone. It can get really easy to be discouraged when someone doesn't come into clinic for a while, and you're really trying to get that follow-up data. Casting a wide net for the collection of, okay, we can collect data on the phone or in person, or we can send the participant a text message to remind them to take a survey. Just really making sure that you're putting all possible methods of communication out 'cause it is difficult to get responses.”* (1W024_ED)

### Theme 4: Research team communication, and communication between research team and clinical team

A recurrent theme during the interviews was the importance of communication between research team members. Some participants stated that this study required more communication than first anticipated:

*“I think it really takes a lot of coordination. I would say more than what we expected. Just from, for example, from building the pain plan, navigating through different hematology providers, and how to make sure everything is also coordinated with other research studies going on. To really nudging the hematologists who may be hesitant in building—who may be not really well-versed with technology and then to trying to understand how to view the pain plan. I think all of those are—we had a great nurse coordinator and was able to be a champion to do that.”* (1W022_ED)

Participants shared that having strong communication between team members was one of the lessons learned while implementing this intervention. The benefits of joint efforts from team members were stressed in multiple interviews:

*“Just having a strong team on the back end to be entering and processing data—because like you said, the soft skills part of it—it's hard to do both. Some people can be really good in person. I am good interfacing with the patients and really enjoy that aspect of it, but then at the end of the day, you do have a lot of data to go through and put, too, so just kind of on the back end, making sure that we have our bases covered. It's really easy to get behind on that data. It adds up”* (1W024_ED)

*“(..) it is a complicated protocol, but it certainly can be done. You need really good communication. You just need good communication. It's between your IT, your research staff, your hematologist, and your ED. That's just what you need is really good communication. You gotta have an ED physician champion, or nothing's gonna happen. I think that's all it really takes really. (…) It's not that hard to do if you're actually committed to doing it.”* (EDP21)

### Theme 5: Challenges with the study protocol

The study protocol is dynamic and requires sites to identify the best procedures for their team and system. In one of the interviews, a participant shared their frustrations with the lack of specific instructions in the protocol:

*“the project did not come to us with a solution. The project came with a "this is what you're supposed to do. Now you need to figure out how to do it in your medical records system.” (..) I think we focused more on the mechanics of doing and the mechanics of data collection than the mechanics of implementation. We've not asked the question, "How do we get more people to use this?" or "How do we get better buy-in?"* (1W028_ED)

Participants spoke about how they thought the eligibility criteria to enroll patients was too restrictive. They found the criteria to be challenging since it eliminated a great pool of patients from being able to enroll in the study:

*“because our priority is that we have an eligibility window of patients had to have some sort of ED visit at the participating ED site for pain crisis in the past 90 days. (..) We have to do pain plans for all of them. If they don't have a pain plan, we still want to enroll them as much as possible. Then think about the details later.”* (1W022_ED)

The protocol required the provider to speak to the patient about their pain plans during their office visit. This procedure takes time which one provider expressed concern about.

“*the point of a plan is to be able to discuss it with a patient, and I'm concerned about adding in another thing to do during the visit. And it may take too much time. And so, we're still working through that one. Yeah, again, some of the providers have that concern.”* (1W035_ED)

### Theme 6: Provider feedback: addressing over-utilizers, patient mistrust, and the positive feedback about the intervention

One of the aims of the provider training video was to address provider perceptions of over utilization of opioids by sickle cell patients. A participant indicated the challenge at the beginning in establishing the protocol because of other physicians’ and ED nurses' perspectives about patients with sickle cell disease:

*“I think that the discriminatory stuff arises almost universally from the fact that providers feel like not enough is being done to address the problem of people who are utilizing, inappropriately, the emergency department. If a provider can go up to a patient and have no concern whatsoever that that person is there for an inappropriate reason, then there is actually not likely to be—unless the person actually believes that (..) people are liars, that it is unlikely that the provider will come into that interaction with a preconceived notion that pain medicine should be withheld. That’s where I find most of the ER doctor bias comes from, that it’s actually—it’s misinterpreted as biased against the patient. What it is, is lack of trust that the provider is willing—that the sickle cell doctor is taking responsibility and taking charge of the problem of inappropriate utilization.”* (1W032_ED)

A participant talked about their experience of presenting the study and the feedback they received. The participant shared a comment that was made by one of the providers after listening to the study proposal:

*“[the person] is African American which was even more surprising to me. [the person] said, “I don’t trust these patients. They lie. They come to the emergency department, they say they have sickle cell disease, they don’t. They lie to you. They use sickle cell disease as an excuse to get the drugs.” (…) Then the other physicians in the room were not supportive. They just heard that comment and they agreed. Then, of course, there was the comment, “Oh, they’re difficult. They come here all the time for pain.” Anyway, that was reflective of the negative perceptions towards our patients.”* (1W037_ED)

*“[physician] was concerned about them abusing their prescriptions, basically. And it concerned about them overusing the Emergency Department.”* (1W036_ED)

Not all the sites shared similar concerns. In one site, a participant mentioned that they “sold” the intervention as a potential solution for provider’s hesitancy:

*“We're: “well, Dr. [physician], don't you think that having a pain plan in the Emergency Department would still be beneficial to them anyway? Even if we couldn't necessarily get to finish the protocol, even if we had a difficult time engaging them or getting them to do the surveys, isn't the whole point in the long run to improve the care for your patient, anyhow?””* (1W036_ED).

One participant described how they addressed concerns from emergency department providers about over-utilizers. These providers were hesitant to use the pain plans for patients they believed over-utilize the department and pain medication. The participant also shared how their response then helped providers gain back trust in patients and the study:

*“We have demonstrated to our ER faculty over the previous decade that when—a few things. Number one, they know very well that we don’t titrate people up on opioids almost ever, that we are aggressively doing everything we can to make sure that the patient is always on the lowest possible amount of opioids that they can be to live as well as they possibly can. Number two, when a patient is inappropriately utilizing the ED, when they’re coming to the hospital too often, when they don’t appear to be having pain, we rapidly investigate, see if there are unmet needs on the patient’s behalf. Are they homeless? (..) You need to do everything that needs to be done to make sure that the ER providers feel that the people who utilize the ED too much are being addressed in other ways, and that this protocol is not designed to make sure that people who over utilize the ED are gonna get lots of doses of opioids. That has to be right at the top, that patients an ultra-utilizer, we’re doing this, this, and that for them. They’ll not be part of this. We are taking care of that in the following ways, and we take responsibility for those patients so that you can rest assured that when you see a pain plan, it belongs there, and it’s the right thing to do for that patient. (…) Try to fix any underlying issues, but if those issues cannot be fixed, we do occasionally move to a situation where our individualized pain plan is not to give opioids to that person in the ED. (…) so our providers trust that when there’s a pain plan that says, “Give opioids,” it’s because the patient should get them (..) It’s just that the ER doctors need to see how hard the hematology team is working to have these patients on good regimens, outpatient, and manage their pain in other ways.”* (1W032_ED)

Another participant shared that they addressed their peers’ concerns immediately and provide reassurance to the emergency department staff that the study was meant to be useful for both patients and providers:

*“It was a question that we had quite a bit when we did our initial provider training, asking about dosage and complaints about people in the population abusing prescription medications. And so we really felt that a large part of this project is reducing stigma in the Emergency Department. So we felt that it was highly important to be upfront with the providers and say, hey, look we know everybody has these cases that they can think of in situations where this behavior happens, but it's not the majority. And it's best practice to not treat it as though it is the majority. So we just, yeah, really felt like addressing that upfront. Could hopefully help reduce some of that stigma and reduce a little of the irritation that some of the providers were feeling.* *(..) really addressing the stigma upfront, I felt also gave us a leg up in that we've already addressed that, that's not what we're here to talk about, we are here to talk about how we get these patients better care in the Emergency Department and how to make it a more streamlined process for everyone. So it's highlighting that this isn't just for our patients, it's just to make your life and your job easier also.” (1W036_ED)*

Importantly, participants reported that patients felt empowered by the pain plans to talk with providers:

*“And the patients also really like the program. They like that they are able to show providers where their pain plan is in the chart. It'll be interesting to see how often that is actually happening when they come to our emergency department, because of course our providers are being guided to the pain plan through the IT intervention and the alert that fires in [EHR]. But the patients are empowered to do it, to use [patient portal] even if they go to another ED that doesn't have our IT supports. And so they really like the idea that any ED provider can be shown their pain plan. And the patients have actually been pretty compliant with the after visit survey.”* (1W033_ED)

The enthusiastic feedback from patients encouraged providers to continue using the pain plans and initiated more buy-in and satisfaction with the study. While reflecting on the study one participant mentioned that a provider who was initially hesitant about the study said:

*"I don't know who came up with these, but boy, that was brilliant." That was good for this particular doctor to hear and [physician] actually wrote back to us and told us about this experience. [The physician’s] words were, "I have to eat crow."* (1W037_ED)

Study teams heard strong positive feedback from the emergency department providers as well:

“*the feedback from faculty has been good about the intervention itself, the IT based intervention. They really like when the patients have a pain plan, an Individualized Pain Plan, they really like having clear guidance on what dose of pain medication should be given when the patient come into the emergency department. So there really has been nothing but positive of feedback because this makes their job easier. They don't have to agonize over it. They also don't have to dig through the chart trying to find the note in hematology that maybe talks about this because now it's always in a specific place in a specific format, they know where to go to find it. And so, there's really been nothing but positive feedback from the providers about the intervention of the pain plans being easily accessible in our EHR when patients come in.”* (1W038_ED)

### Theme 7: COVID-19 and its effects on study implementation

The pandemic challenged the implementation of the study. Participants talked about how the pandemic made the process of recruiting patients more difficult since patients are avoiding the emergency department and their office visits. People are avoiding the ED to avoid COVID exposure, and they prefer telehealth, but sometimes they also cancel their telehealth visit. “*It’s just hard to reach people if you’re tryin’ to contact them at home. I just feel like in person is so much better to have their attention.”* (1W027_ED). Another provider mentioned the avoidance of emergency department: “*I've had patients say, "Well, I don't want to go to the ED because of COVID. There are a lot of COVID patients there, and I don't want to go."* (1W038_ED).

Since patients were avoiding the hospital and switching to telehealth, coordinators had to switch their in-person recruitment methods to remote ones. A participant discussed their experience navigating this shift:

*“I think because of COVID, we learned a lot about approaching people by phone and because I would've guessed it would've been very challenging to recruit patients for this study by phone, but actually it's worked quite well. (…) I think many of them also discovered telemedicine and said, "Nice, don't have to leave my house and come to the clinic." And they can just do a tele-visit with their provider. There are times though when they need to come in because there are blood tests or there's something they have to come do in person, but there are many fewer in-person clinic visits than there were before COVID. So we really had to adjust our recruitment strategies to account for the fact that patients were not coming very regularly to clinic.”* (1W033_ED)

Due to the pandemic, telehealth visits with hematologists have become a more common practice. That presented a challenge for the research team as it made the process of tracking patients and contacting them more difficult. This difficulty was expressed by participants during the interviews:

*“The other is that a telemedicine visit is a different flavor. (…) keeping an agenda is a little bit more complicated for the telemedicine visit. We have to talk more ‘cause we can’t get as much nonverbal or physical exam.*”

## Discussion

This study aimed to understand the process of implementing an E-IPP for emergency department treatment of vaso-occlusive episodes in adults with SCD. The ALIGN study was complex in its implementation. Participants mentioned the challenges in developing the E-IPP, including communication with the informatics team and defining how to make the E-IPP visible for physicians in the emergency department. At the beginning of this project, several sites having to develop the plan and embed it in their electronic health records (EHRs) as well as embedding them in the EHR’s patient portal application [25]. While time was budgeted to survey hospitals about their EHRs, and templates were created to support the development of IPPs, participants overwhelmingly shared about the challenges that involved communicating and working with their informatics department. A lesson learned from this study is the early inclusion of IT personal and leadership as study team members to support the development of the EHR platform.

The role of the ED champions was a common thread across sites. The champions had important roles in sharing the value added of the study with their peers, as well as in addressing potential stigma and discrimination of their peers towards patients with SCD. The concept of champions to support the implementation of healthcare interventions has garnered importance in the literature, albeit with different names (e.g., champion, knowledge broker, change agent) [32, 33]. Participants shared the importance of someone knowledgeable about the organization and with institutional and social power who was able to “sell” the study to the peers. For some sites, this person was identified after the study was launched and sites were having a hard time with providers answering the surveys; for other sites the ED champion was part of the study team from the beginning. All participants, however, verbalized the importance of early engagement of the champion early on in the study. It will be important for study sites in the future to clearly identify the skills needed for the champion as part of their implementation strategy [34] to increase the probability of success.

The study protocol was complex. In other words, the patients’ eligibility criteria (an ED visit within the past 90 days) demanded high coordination from the study coordinators and clear communication among team members. Communication, trust and relationship have been shown to be important factors in “implementation teams.” An implementation team can be defined as the team in charge of designing and leading the implementation of an intervention [35], and there is some evidence indicating that teamwork predicts the success of implementation [35, 36]. An overarching theme in this study was the importance of coordinators, who often had high trust and respect from the Principal Investigators, patients, and clinic staff to lead and coordinate the study. In fact, the literature on implementation practitioners indicate the importance of network and of skills such as interdependence, and collaborative practice [37, 38]. It is worth noting that the restrictive eligibility criteria were necessary to recruit the patients most likely to visit the ED and achieve the required number of events to evaluate the intervention in the ALIGN Study. Outside the constraints of a research study, the intervention could be offered more broadly.

Topics such as racism and resistance of some providers towards the study with the perception that patients could overuse opioids was explicit in some interviews. Racism towards SCD patients in emergency departments is not new [13]. Often, providers may not be aware of how their practices affect the care of patients with SCD in a busy, chaotic and complex environment such as the emergency departments [39]. The participants from this study indicated strategies to address these challenges, such as addressing racism right at the beginning when explaining the study, providing an outline that describes how hematologists would address potential over users such as connecting patients with resources to address social determinants of health or untreated mental health conditions, and sharing patient and other providers’ perspectives about the study. As a response to some of these strategies, a participants shared: “*I've even had a provider respond to me after completing the survey and said, "Thank you so much for doing this. This makes caring for this population effortless."*(1W036_ED). As noted above, ED providers also liked the intervention because it supported their practice in providing quality care for their patients with SCD. We should note that only patients who had a hematologist to create an IPP were included in the study, so SCD patients who are high utilizers in the ED due to lack of access to hematology care were not addressed in this intervention. In such cases, affiliation with a sickle cell specialty provider would likely be the first major step to improving care [40].

Some of these challenges were enhanced with the COVID-19 pandemic when patients were less likely to visit emergency departments. As with many studies, coordinators had to learn alternative methods to recruit patients, including phone calls, emails, and use of the hospital’s telehealth platform. While challenging, some participants shared that some patients appreciated the telehealth as that allowed them to connect with their provider without having to travel.

### Implications for clinical practice

The implementation of IPP’s in the EHR for both ED providers and in the patient portal requires champions and partnerships with hematology, emergency medicine, nursing and informatics. While there are challenges with the initial infrastructure (i.e., development of the E-IPP and embedding it in the EHR) and in the process (i.e., implementation team coordination and communication; gathering “buy-in” from different clinicians), there was overall success with implementation at each center and we learned many lessons that can help other institutions. The complexity of the intervention can be overcome by planning the pre-implementation phase of the study, by identifying key team members from EM, hematology and informatics, pre-empting concerns, and planning strategies that can support the intervention. Several participants shared that, despite the challenges, patients and providers really liked the intervention: patients are empowered to use their portal, and providers can easily find the information that they need. Such positive feedback has indeed been reported in surveys with patients and physicians [41]. Table 1 shows the identified barriers and potential solutions based on the interviews of this study, which can be used to inform future work. While measurement of study outcomes added to the complexity of the protocol, implementing the E-IPP for clinical care alone would be much less complex once the infrastructure of the EHR and communication among providers and patients is established.

**Table 1:** Challenges and facilitators identified by the participants, per theme

	Challenges	Facilitators
Theme 1: The IPP structure (location, format, and upkeep) and collaboration with the informatics team	- Finding a place for the IPP that is easy to find	- Early and close collaboration with the informatics team
	- Building the IPP in different electronic record systems	
	- Updating IPP	- Develop a template for IPP that can be adapted by sites
		- Develop a plan with hematologists and clinic coordinators to update IPPs
		- Have simple IPP, with opioid dosing, SCD provider contact and genotype
		- Help ED docs and nurses to see that the IPP can help speed their workflow
		- Put the IPP in the health record where a Patient Portal can access it—a patient showed their IPP to a new ED when traveling out of state and that ED acted upon the IPP as credible information closely collaborate with IT and ED physicians to identify where is best to host the IPP. In some sites, it was stored in the FLAG section of the EPIC electronic medical record so that it easier for ED providers to find than in hematology progress notes
Theme 2: The role of ED champion	- Power differential among different members of the clinic can make buy-in of study difficult	- Identify an ED champion that can “sell” the importance of IPP
	- Developing IPP from scratch can entail a lot of work for hematologists, who may not want to participate in the study	- Develop templates for hematologists to support development of IPP
		- Share positive feedback from patients and other clinicians about the value added of the study
Theme 3: The role of coordinators	- Complex protocol, study coordinators have to track several moving pieces (e.g., inclusion criteria, post ED visit survey)	- Develop clear communication paths; clear roles for coordinators and other members of the study (e.g., nurse coordinator)
	- Difficulty recruiting patients	- Create multi-modal recruitment strategies
		- Have weekly check in meetings with research team
Theme 4: Research team communication and dynamics	- Protocol requires a lot of people (e.g., study coordinators, nurses, hematologists, ED clinicians) to be successful	- Facilitate communication and trust among different team members
		- Identify different strengths (e.g., good at interacting with people, good at data tracking) and capitalize on strengths
Theme 5: Challenges with the study protocol	- Inclusion criteria is challenging (ED visit in the past 90 days)	- Ensure that all patients have IPP and MyChart to facilitate recruitment
Theme 6: Provider resistance: addressing over-utilizers, and patient mistrust	- Some physicians may believe that patients are misusing or abusing opioids	- Engage with ED physicians early in the process and explain the role of IPP and how hematologists may address over-use of opioids
		- Engage with an ED champion who may be able to talk with peers
		- Address the stigma upfront and early in the study
Theme 7: Covid-19 and its effects on study implementation	- SARS COVID-19 affected recruitment of patients, as most avoided going to the ED	- Learn different skills on how to recruit and engage patients over the phone or via the hospital telemedicine platform
	- Patients started engaging with telemedicine	
		- Develop multi-model recruitment materials to engage patients

Since study completion, the National Alliance of Sickle Cell Centers posted a research-based, opioid and weight-based calculator for use by individual SCD providers. The calculator guides SCD providers in determining an ED IV opioid dose based on acute and chronic pain history of the patient, (https://sicklecellcenters.org/provider_resources). A second resource was developed by the Sickle Cell Disease Association for America in partnership with MedicAlert. The current plan is for the company to store IPPs online and provide a bracelet and a wallet card with QRcode to have the information available, with the goal of making the IPP credible and supporting its uptake by ED providers. Finally, the American College of Emergency Physicians developed a decision support, Point-of-Care-Tool (website and mobile app) to guide sickle cell acute management in the ED. The DST follows recommendations from the National Heart, Lung and Blood Institute (NHLBI) and American Society of Hematology (ASH) guidelines and supports the use of IPP’s as first choice, and weight-based dosing as second choice.

### Limitations and strengths of the study

This study aimed to examine the implementation process of the SCID ALIGN study. Due to the pandemic, interviews were conducted via zoom and as such some nuances about team dynamics and non-verbal cues could have been missed. Nevertheless, participants were engaged in the interviews and member checking was used to ensure integrity of the data. This study allowed us to learn about the implementation of the protocol but not sustainability beyond about 1 year. The outcome was limited to use of the IPP, not to a patient-centered outcome or to a healthcare utilization outcome. Future research questions may entail examining the sustainability of E-IPPs in the sites as well as the development and testing of strategies to scale up this intervention to other sites. We also restricted the data collection to gather information from the implementation team; a 360 approach capturing the perspectives from the ALIGN participants would enhance the contextual understanding of this study. Accordingly, a separate study is collecting data from the ALIGN participants’ perspective.

## Conclusion

Findings from this study contribute to learning how to implement E-IPPs for adult patients with SCD in emergency departments. Early engagement with different team members, including informatics team, a champion from the emergency department, study coordinators with different skills and enhancement of communication and trust among team members may be important for the success of this protocol.

### Supplementary Information

**Additional file 1: Appendix 1. **Baumann SCDIC Tracking Strategies – ED Protocol.

## Data Availability

Data can be requested from the corresponding author on reasonable request.
