# Supplementary material for: “The project did not come to us with a solution”: Perspectives of research teams on implementing a study about electronic health record-embedded individualized pain plans for emergency department treatment of vaso-occlusive episodes in adults with sickle cell disease
Source: BMC Health Serv Res. 2023 Nov 13;23:1245. doi: 10.1186/s12913-023-10255-7 (PMC10641983; doi:10.1186/s12913-023-10255-7)
Supplement: Supplementary file 1 — Additional file 1: Appendix 1. Baumann SCDIC Tracking Strategies – ED Protocol. [file 12913_2023_10255_MOESM1_ESM.docx]

**Appendix 1: Baumann SCDIC Tracking Strategies – ED Protocol**

**Baumann SCDIC Tracking Strategies – ED Protocol**

*(NOTE: the protocol was iterated across interviews to enhance quality of the questions based on the experience of the sites)*

**Aims**

The overall purpose of this project is to learn about the implementation strategies, or the processes that will support the implementation of the interventions developed by the Sickle Cell Disease Implementation Consortium (SCDIC).

**Script**

Good morning/afternoon. Thank you for meeting with me. The goal of this study is to capture the implementation strategies, or the processes that will support the implementation of the interventions developed by the Sickle Cell Disease Implementation Consortium (SCDIC). This study has been approved by the WU IRB.

I will interview three people from your site. The information shared in this call will be treated with confidentiality and will not be shared with your peers from your site or other sites; however people from your site may know that you have participated in this interview.

You can end this conversation at any time with no consequences. No one will know how long we spoke or if you ended the conversation early or declined to or were unable to answer any questions.

This conversation will be recorded. If you have confidential information that you would prefer not to be recorded, let me know and I can pause the recording.

Do you have any questions?

Can I start recording?

Today, we will be talking about the Emergency Department Protocol.

As a reminder, the Emergency Design protocol aims to improve management of vaso-occlusive episodes (VOEs) in adult EDs by embedding Individualized Pain Plans (IPPs) in the electronic health record (EHR).

We are interested in examining how your team will facilitate the implementation of these interventions in your site. I’ll ask about the implementation strategies for the providers and patients separately.

***Providers***

Let’s first talk about the providers. As you know, Providers will only count as enrolled if they have completed the baseline survey.

- How are you recruiting providers?
- Who is recruiting them?
- How often?
- What are some of the challenges and some of the things that are helping in recruiting the providers?

***Patients***

Now let’s talk about the patients. Let’s first talk about recruitment.

- How are you recruiting patients?
- Where?
- Who is recruiting them?
- What is the training of the person doing recruitment?
- What are some of the challenges in recruiting them?
- What are some things that are helping in recruiting patients?

Are you doing anything to maintain their engagement in the study?

What are the extent to which these strategies can be adapted?

- 1. What can be adapted? (e.g., frequency, mode of delivery)
  2. On what basis can the strategies be adapted? (e.g., participant progress, spontaneity, checklist)
  3. When can the strategies be adapted? (e.g., when delivering the strategy, adaptations need to be discussed with the team)

How confident are you that the strategies to support these interventions that you are planning will work? How are you assessing whether these strategies work?

Thank you. Now let’s talk about your team. Implementing a study involves a lot of decisions and logistics. How are decisions being done?

- Who is making the decisions about the strategies that you are planning to use?
- How do you communicate with each other to support the implementation of this study?

Do you have any further comments about the processes that you and your team are going through in planning the strategies to implement the ED protocol?
